# Dissociative Experiences and Psychopathological Symptoms in a Non-Clinical Sample—Gender Differences and the Mediating Role of Self-Concept Clarity

**DOI:** 10.3390/ejihpe16050064

**Published:** 2026-05-03

**Authors:** Georgiana Bogos, Octav-Sorin Candel, Ana Tiperciuc, Magdalena Iorga

**Affiliations:** 1 Faculty of Psychology and Educational Sciences, “Alexandru Ioan Cuza” University of Iași, 700554 Iasi, Romania; bogosgeorgiana@gmail.com (G.B.); octav.candel@uaic.ro (O.-S.C.); ana_tiperciuc@yahoo.com (A.T.); 2 Department of Behavioral Sciences, Faculty of Medicine, “Grigore T. Popa” University of Medicine and Pharmacy, 700115 Iasi, Romania

**Keywords:** dissociative experiences, psychopathological symptoms, self-concept clarity, anxiety, depression, somatization, interpersonal sensitivity

## Abstract

Background: Dissociation is associated with multiple psychiatric disorders and mental health issues. However, there are some limitations in the existing studies, such as the predominant use of clinical samples and the lack of focus on potential mediators that can explain this relationship. Among the latter, an unclear self-concept may serve as a risk factor for many psychological disturbances, while a well-established self-concept may be a resilience factor. This study aimed to examine the gender differences among the variables, to test whether dissociative experiences are associated with psychopathological symptoms in a non-clinical sample, and to explore the mediating role of self-concept clarity. Materials and methods: 257 participants (*M_age_* = 23.92 ± 7.33, 69.6% females) were included in the research. The Symptoms Checklist-90, The Dissociative Experiences Scale, and The Self-Concept Clarity Scale were used. Results: Women scored higher on somatization (*M_women_* = 1.28, *SD* = 0.86) than men (*M_men_* = 0.93, *SD* = 0.70), *t*(255) = 3.47, *p* = 0.001, *d* = 0.42. Moreover, women had a higher level of depression (*M_women_* = 1.37, *SD* = 0.96) compared to men (*M_men_* = 0.98, *SD* = 0.77), *t*(255) = 3.49, *p* = 0.001, *d* = 0.43. Dissociative experiences were positively associated with psychopathological symptoms (*r* = 0.69, *p* < 0.01). Self-concept clarity mediated this relationship (*β* = 0.21, *SE* = 0.04, 95% CI [0.13, 0.30]). Conclusions: The findings showed gender differences regarding psychopathological symptoms and highlighted the importance of self-concept clarity in reducing the risk of developing them.

## 1. Introduction

Dissociation is a defense mechanism in which conflicting impulses are kept apart or threatening ideas and feelings are separated from the rest of the psyche (VandenBos, 2015). Since dissociative experiences are cognitive processes manifested by a variety of symptoms, they can occur in both clinical and general populations (Mhanna et al., 2022). First, dissociation occurs as a symptom in almost all psychiatric disorders (e.g., borderline personality disorder, post-traumatic stress disorder, anxiety and affective disorders, schizophrenia, etc.) (Lyssenko et al., 2018). Second, some symptoms of dissociation, such as absorption and amnesia, can also be observed in the general population (Kate et al., 2019). Nevertheless, a well-established self-concept may be a resilience factor associated with decreased levels of psychopathology caused by dissociative experiences (Lassri et al., 2023). These findings highlight the importance of studying dissociative experiences not only in clinical populations, but also in non-clinical populations, since they are considered a risk factor for self-destructive and even suicidal behaviors (Loewenstein, 2018).

Dissociation is conceptualized as a defense mechanism that helps people affected by trauma or extreme stress to detach from negative emotions in order to protect the psyche, but in the long term it can contribute to a sense of emotional detachment, difficulties in regulating emotions, anxiety associated with panic attacks, and somatization (Hozoori & Barahmand, 2013; Ural et al., 2015). Mild forms of dissociation are common in the general population. However, even when mild, they can occur alongside depression (Fung et al., 2022).

In severe forms, the phenomenon of dissociation has been observed as detachment and compartmentalization (Holmes et al., 2005). Detachment refers to a feeling of separation from oneself (depersonalization) or from the external world (derealization), while compartmentalization represents a separation of memories and emotions from consciousness (Holmes et al., 2005; Van der Hart, 2021). In accordance with the continuum model (Braun, 1988), dissociative experiences can be experienced in both pathological and non-pathological forms, so it is essential to distinguish between these two forms. When dissociation occurs as a response after exposure to severe stress, it can become pathological and can negatively interfere with professional and social functions (Chiu et al., 2017; Schimmenti, 2016). In contrast, absorption and hypnotic states are non-pathological phenomena (Butler et al., 2019). Considering the complexity of manifestations, focusing on non-pathological symptoms would potentially contribute to the literature.

Non-clinical studies have suggested non-significant gender differences in terms of dissociative symptoms (Luoni et al., 2018; Spitzer & Freyberger, 2008). However, other studies indicate that women report significantly higher scores on derealization and depersonalization, while men report significantly higher scores on amnesia compared to women (Spitzer et al., 2003). These differences could be explained by the fact that women tend to present higher emotional reactivity and a stronger tendency towards internal processing, which favors experiential forms of dissociation (depersonalization, derealization), while men are more inclined towards avoidant coping styles and emotional suppression (Engel et al., 2021; Stoica et al., 2021).

People with a high level of dissociation respond less effectively to treatment for depression and anxiety and are more likely to develop obsessive–compulsive symptoms compared to people with a low level of dissociation (Boysan, 2014; Prasko et al., 2016). Therefore, dissociative experiences may be linked to psychological symptoms, largely due to difficulties in managing emotions and because they impair the perception of self-identity (Faustino et al., 2025).

Self-concept clarity refers to how clearly and confidently a person’s self-concept is defined, how internally consistent it is, and how stable it remains over time (Campbell et al., 1996). In essence, the self-concept clarity is based on introspection leading to a state of certainty or uncertainty regarding the self (Paetzold & Rholes, 2021).

According to identity integration theory (Syed & McLean, 2016), the way in which a person organizes the various aspects of their identity (e.g., roles, values, emotions) into a coherent and stable sense of self highlights that the degree of coherence and consistency in self-representations is essential for understanding the internal organization of identity and adaptive psychological functioning. From this viewpoint, the clarity of the self-concept could be considered an explanatory mechanism through which dissociative experiences are related to psychological symptoms, since dissociation may fragment and destabilize self-representations, which, in turn, may contribute to increased psychological distress.

When it comes to gender differences in self-concept clarity, some studies have found no differences, while others have suggested that women have lower self-concept clarity than men because women tend to identify themselves through their interpersonal relationships (Cicero, 2020; Chen et al., 2006). Additional explanations might be based on the fact that women tend to ruminate more and are more sensitive to social feedback than men (Birditt et al., 2024; Tobias & Ito, 2021).

Mental wellbeing is closely related to clarity of the self-concept (Hong et al., 2022). In the study conducted by Coutts et al. (2023), self-concept clarity was positively associated with high life satisfaction but also with lower symptoms of perceived stress and depression. Previous studies further support that a low self-concept clarity may be associated with a range of psychopathologies, including anxiety, depression, psychotic spectrum disorders, as well as more maladaptive psychological functioning (Cicero, 2017). As a consequence, individuals may tend to view life as chaotic, stressful, and unpredictable (Hong et al., 2022). This finding may attest that coping style also has an important role in developing a stronger self.

On the one hand, as in the case of dissociative experiences, traumatic episodes are a risk factor because they can disrupt the development of an integrated self-concept, which leads to a lower self-concept clarity and consequently to a higher vulnerability for the development of psychotic symptoms (Cicero et al., 2016; Cicero, 2017; Evans et al., 2015). On the other hand, a clear and stable sense of self overtime substantially contributes to psychological adjustment and can help people cope with stressors that are perceived as uncontrollable or threatening, and at the same time may have a protective role against dissociative experiences (Lee-Flynn et al., 2011; Paetzold & Rholes, 2021).

Psychopathological symptoms refer to behavioral changes (e.g., withdrawal from activities), thought disturbances (e.g., detachment from reality), and emotional changes (e.g., mood swings) that are specific to mental disorders (Parnas et al., 2013). The diathesis–stress model (Monroe & Cummins, 2014) suggests that psychopathological symptoms result from the interaction between an individual’s internal vulnerabilities (e.g., genetic predisposition, increased emotional sensitivity) and external stressors (e.g., trauma, frequent conflicts), with this interaction leading to the manifestation of specific psychological disorders. From this perspective, dissociative experiences may be understood as indicators of an underlying vulnerability, specifically decreased self-concept clarity. This deficit can compromise a person’s ability to control and interpret their inner states, therefore increasing the likelihood of psychopathological symptoms.

The Hierarchical Taxonomy of Psychopathology model supports the organization of symptoms into broad dimensions (e.g., externalizing symptoms, internalizing symptoms, psychotic thinking), highlighting the fact that some symptoms can be combined and thus overlap between different disorders (Kotov et al., 2018). This approach is supported by previous research showing the existence of a psychopathology factor that is associated with common cognitive and emotional vulnerabilities (Haywood et al., 2022). In this context, the literature emphasizes the existence of common explanatory processes that contribute to the organization of psychopathological symptoms. Thus, a reduced clarity of the self-concept can be understood as a central mechanism reflecting difficulties in organizing and integrating psychological experience, while dissociation is associated with psychopathology through its relationship with this self-disorganization (Hertel et al., 2024; Paetzold & Rholes, 2021).

Furthermore, psychopathological symptoms may differ depending on gender. Data from previous studies suggests that, compared to males, females report significantly higher scores regarding anxiety, phobic anxiety, depression, interpersonal sensitivity, somatization, and the total score of psychopathological symptoms (B. Carter et al., 2022; Landazabal et al., 2008). These differences could be explained by a combination of biological, cognitive, as well as sociocultural factors, including increased emotional reactivity, a greater tendency toward rumination, and a more intense perception of psychological distress among women compared to men (Birditt et al., 2024). Other studies have indicated evidence that, in general, females experience more internalizing symptoms (e.g., anxiety, depression, somatization) while males experience more externalizing symptoms (e.g., aggression, conduct disorder) (Hoffmann et al., 2004).

Considering the major role of psychopathological symptoms in the structure and function of the self, self-concept clarity has been proposed in previous studies as a mediator in the relationship between self-compassion and depressive symptomatology (Coutts et al., 2023) and also in the relationship between trauma and psychosis (Evans et al., 2015). Previous studies have also suggested that low self-concept clarity acts as a mechanism that leads to the onset of anxiety and depression in young adults (Hertel et al., 2024). However, it has not yet been proposed as a mediator in the relationship between dissociative experiences and psychological symptomatology.

The present study aimed to examine whether there are gender differences in terms of dissociative experiences, psychopathological symptoms, and self-concept clarity. We also aimed to identify to what extent psychopathological symptoms are associated with dissociative experiences and self-concept clarity in a non-clinical sample. A final aim was to examine whether there is a mediating role of self-concept clarity in the relationship between dissociative experiences and psychopathological symptoms. Based on the fact that dissociation disrupts the integration of cognitive and emotional experiences, leading to the fragmentation of self-representations and, consequently, to a diminished sense of self (Chiu et al., 2017), we could further assume that this reduction in the coherence and stability of the self-structure may compromise the individual’s ability to interpret and self-regulate internal experiences, thereby constituting the mechanism through which dissociation is associated with the onset of psychological symptoms. Therefore, unlike other plausible mediators, a low self-concept clarity is a mechanism more closely linked to the processes of identity disintegration (M. J. Carter & Bruene, 2019).

Based on the previous literature presented above we anticipated that:

(1) There are differences in dissociative experiences, psychopathological symptoms, and self-concept clarity according to gender, with higher scores in women than in men regarding psychopathological symptoms and dissociative experiences, but with lower scores in terms of self-concept clarity compared to men;
(2) There is a negative association between dissociative experiences and self-concept clarity;
(3) There is a negative association between self-concept clarity and psychopathological symptoms;
(4) The link between dissociative experiences and psychopathological symptoms is mediated by self-concept clarity. Specifically, we anticipated that higher levels of dissociative experiences will be associated with lower self-concept clarity, and decreased self-concept clarity will be associated with increased psychopathological symptoms.

## 2. Materials and Methods

### 2.1. Participants and Procedure

The sample of our study included 257 participants from the general population, aged between 18 and 61 years old (*M* = 23.92, *SD* = 7.33). A series of Monte Carlo power simulations targeting indirect effect sizes with bootstrapped confidence intervals showed that the sample was sufficient to detect significant indirect effects at an adequate power of 0.80 (Schoemann et al., 2017). Individuals who agreed to participate were informed about the objective of the study and they were assured of the confidentiality and anonymity of their responses. They were also told that all data provided would only be used for research purposes and that they could withdraw at any time, without any consequence. Then, the participants were asked for their consent, provided their socio-demographic data, and filled in the questionnaires measuring psychopathological symptoms, dissociative experiences, and self-concept clarity. For each scale, clear instructions were provided on how to answer the items. The time needed to complete the form was around 15 min. Participants were not rewarded, but they were informed of the possibility of receiving a personalized report of their responses and were given a gratitude message at the end of their participation.

Participation was voluntary and the inclusion criteria were (1) age of at least 18 years and (2) completion of the questionnaire before the deadline. After eliminating any diagnosis of a mental disorder, a total of 257 respondents were included in the research.

### 2.2. Instruments

To collect data from the participants, we created an online form, which was distributed on several social media platforms, through the snowball technique, during the period between 12 and 26 February 2026. Therefore, no specific strategies were implemented to ensure sample representativeness. Participants were asked to provide their age, gender, place of origin (urban/rural), last educational level achieved, whether they had any health problems in the last year and, if so, what the diagnosis was, and whether they suffered from any chronic illness and, if so, what the diagnosis of the chronic illness was.

Dissociative experiences were assessed using *The Dissociative Experiences Scale* (DES-II; Carlson & Putnam, 1993). This scale has 28 items, measuring dissociation as a trait, namely, the frequency of people’s dissociative experiences (e.g., “Some people have the experience of looking in a mirror and not recognizing themselves. Circle a number to show what percentage of the time this happens to you.”). Each item is assessed on an 11-point Likert scale, ranging from 0 (*never*) to 100 (*always*). Although initial studies analyzing the factorial structure of the scale proposed three dimensions (*amnestic dissociation*, *absorption and imaginative involvement*, and *depersonalization/derealization*) (e.g., Carlson et al., 1991), no consensus has been reached regarding the factors of the scale (Lyssenko et al., 2018). Therefore, the present research used the total score, considered by the authors of the scale to be the only reliable measure (Carlson & Putnam, 1993). Higher scores indicate higher levels of dissociative experiences. Considering that, in non-clinical samples, certain subtypes of dissociation may be underrepresented, thereby limiting differential analyses (see Mazzotti et al., 2016), we used the total score of dissociative experiences to assess the risk associated with psychopathological symptoms. The scale showed its psychometric properties both in clinical and non-clinical samples and across different cultures (e.g., Carlson & Putnam, 1993; Oh et al., 2015; Schimmenti, 2016), including Romanian samples (e.g., Bogos et al., 2026; Vancea & Apostol, 2021). Cronbach’s alpha coefficient in our study was α = 0.96, which is similar to the findings reported by Faustino et al. (2025).

*The Self-Concept Clarity Scale* (SCCS; Campbell et al., 1996) was used in order to measure self-concept clarity as a trait. This unidimensional scale consists of 12 items rated on a 5-point Likert scale, ranging from 1 (*strongly disagree*) to 5 *(strongly agree*). The items assess the degree to which one’s sense of self is well-defined, stable in time and consistent (e.g., “I seldom experience conflict between the different aspects of my personality”). The total score (the mean value of the items) was computed after the ten reverse items were recorded (all of them, except 6 and 11), with higher scores representing higher levels of self-concept clarity. The scale has shown adequate psychometric properties in non-clinical samples (e.g., Coutts et al., 2023) and was used in Romanian research as well (e.g., Cicei, 2012; Isbăşoiu et al., 2021). In the present research, Cronbach’s alpha was α = 0.79. Previous studies also sustain the reliability of the scale (e.g., Yang et al., 2025).

Psychopathological symptoms were evaluated using *The Symptoms Checklist-90* (SCL-90; Derogatis et al., 1973), an inventory designed to assess various symptoms of psychopathology. It is composed of 90 items, measuring the frequency of each symptom and rated on a Likert scale from 0 (*not at all*) to 4 (*extremely*). The items are distributed across nine dimensions: *somatization* (e.g., “A lump in your throat”), *obsessive–compulsive* (e.g., “Trouble concentrating”), *interpersonal sensitivity* (e.g., “Feeling inferior to others”), *depression* (e.g., “Feeling blue”), *anxiety* (e.g., “Trembling”), *anger–hostility* (e.g., “Getting into frequent arguments”), *phobic anxiety* (e.g., “Feeling afraid you will faint in public”), *paranoid ideation* (e.g., “Feeling that you are watched or talked about by others”), and *psychoticism* (e.g., “Hearing voices that other people do not hear”). In the present research, we used both the total score, represented by the Global Severity Index (GSI)—the average of all items—and the scores on each dimension—the mean value of their items. Higher scores indicated higher levels of psychopathological symptoms. This scale was used not only in clinical settings, but also in non-clinical ones, with people from the general population (e.g., Gualtieri et al., 2025) and with Romanian samples (e.g., Albai et al., 2021; Enatescu et al., 2021; Popescu et al., 2024). In our study, Cronbach’s alpha coefficient was α = 0.98 for the total scale, α = 0.90 for *somatization*, α = 0.85 for *obsessive–compulsive*, α = 0.88 for *interpersonal sensitivity*, α = 0.92 for *depression*, α = 0.91 for *anxiety*, α = 0.87 for *anger–hostility*, α = 0.82 for *phobic anxiety*, α = 0.84 for *paranoid ideation*, and α = 0.85 for *psychoticism*. Previous studies also reported good psychometric properties in non-clinical samples (Du et al., 2022).

### 2.3. Statistical Analysis

First, using the SPSS 26 software, we conducted descriptive preliminary analyses to compute means, standard deviations, minimum and maximum scores, and to check the normality distribution of data. We used independent samples *t*-tests to analyze the gender differences in dissociative experiences, self-concept clarity, and psychopathological symptoms (total score and the specific dimensions). Next, correlations between the study variables were computed. We performed mediation analyses using the PROCESS extension for SPSS, version 4.2 (Hayes, 2022). Model 4 was applied, allowing us to test the mediating role of self-concept clarity by using 5000 bootstrap samples to estimate 95% confidence intervals. The indirect effect (mediation) was considered significant if the confidence interval did not contain zero.

## 3. Results

### 3.1. Descriptive Statistics

The socio-demographic characteristics of the participants are reported in Table 1.

In accordance with the inclusion criteria described below, participants who reported having suffered from a mental health problem (such as depression, anxiety, obsessive–compulsive disorder, borderline personality disorder, or schizophrenia) were excluded from the study. Among the medical diagnoses reported by the participants in our final sample as being received in the last year, there were: metatarsian fracture, spinal fracture, spondylosis, Hashimoto’s hypothyroidism, tubo-ovarian abscess, iron deficiency anemia, gastroesophageal reflux disease, psoriasis, urinary infection, and double herniated disc. Among the chronic illness diagnoses, the following were reported: biliary dyskinesia, scoliosis, atrial septal defect, temporal lobe epilepsy, minor systolic murmur, and food intolerances.

All skewness and kurtosis values were between −2 and 2, as suggested by George and Mallery (2010), so the data in our sample was normally distributed (see Table 2).

### 3.2. Comparative Statistics

Multiple pairwise comparisons were conducted using independent samples *t*-tests. To control for Type I error, a Bonferroni correction was applied, resulting in an adjusted significance threshold of a = 0.001.

The gender differences in the study variables are reported in Table 2.

The independent samples *t*-test showed non-significant gender differences in dissociative experiences, self-concept clarity and the SCL-90 total score. Moreover, there were non-significant differences in SCL-90 dimensions, with all *p*s > 0.001, except for s*omatization*, *t*(255) = 3.47, *p* = 0.001, *d* = 0.42, and *depression*, *t*(255) = 3.49, *p* = 0.001, *d* = 0.43. Women present higher levels of *somatization* (*M* = 1.28, *SD* = 0.86) and *depression* (*M* = 1.37, *SD* = 0.96) than men (*M* = 0.93, *SD* = 0.70; *M* = 0.98, *SD* = 0.77).

Based on residential area, the independent samples *t*-test revealed non-significant differences in dissociative experiences, self-concept clarity, SCL-90 total score, and its dimensions, with all *p*s > 0.001.

In terms of educational level, participants were divided into two categories: pre-university and university education. The independent samples *t*-test revealed non-significant differences in dissociative experiences and self-concept clarity, with all *p*s > 0.001. However, there were significant differences in the total score of psychopathological symptoms, *t*(255) = 3.29, *p* = 0.001, *d* = 0.40, as well as in the following dimensions: *interpersonal sensitivity*, *t*(255) = 3.30, *p* = 0.001, *d* = 0.40; *hostility*, *t*(255) = 3.29, *p* = 0.001, *d* = 0.40; *paranoid ideation*, *t*(255) = 3.84, *p* < 0.001, *d* = 0.49; and *psychoticism*, *t*(255) = 3.81, *p* < 0.001, *d* = 0.54. The participants with pre-university education report higher levels of overall psychopathological symptoms (*M* = 1.24, *SD* = 0.79) compared to participants with university education (*M* = 0.94, *SD* = 0.67). Furthermore, the participants with pre-university education report higher levels of *interpersonal sensitivity* (*M* = 1.45, *SD* = 0.96), *hostility* (*M* = 1.10, *SD* = 1.02), *paranoid ideation* (*M* = 1.40, *SD* = 0.94), and *psychoticism* (*M* = 0.93, *SD* = 0.60) compared to participants with university education (*M* = 1.09, *SD* = 0.77; *M* = 0.72, *SD* = 0.84; *M* = 0.96, *SD* = 0.84; *M* = 0.60, *SD* = 0.61).

### 3.3. Correlation Analysis

To verify the associations between the study’s variables, Pearson correlations were computed (see Table 3). The results revealed that dissociative experiences were significantly and negatively associated with self-concept clarity and positively correlated with psychopathological symptoms (SCL-90 total score and all of its dimensions).

A significant negative relation between self-concept clarity and psychopathological symptoms (both for SCL-90 total score and its individual dimensions) was registered.

Age showed a significant negative association with dissociative experiences, the psychopathological symptoms total score, and all of the above-mentioned dimensions. In contrast, a significant positive correlation was found between age and self-concept clarity.

Collinearity diagnostics were conducted to assess potential issues of multicollinearity among the predictors. The results indicated no multicollinearity concerns, with all VIF values within acceptable limits (VIF < 2).

### 3.4. The Mediating Role of Self-Concept Clarity

**Figure 1:**
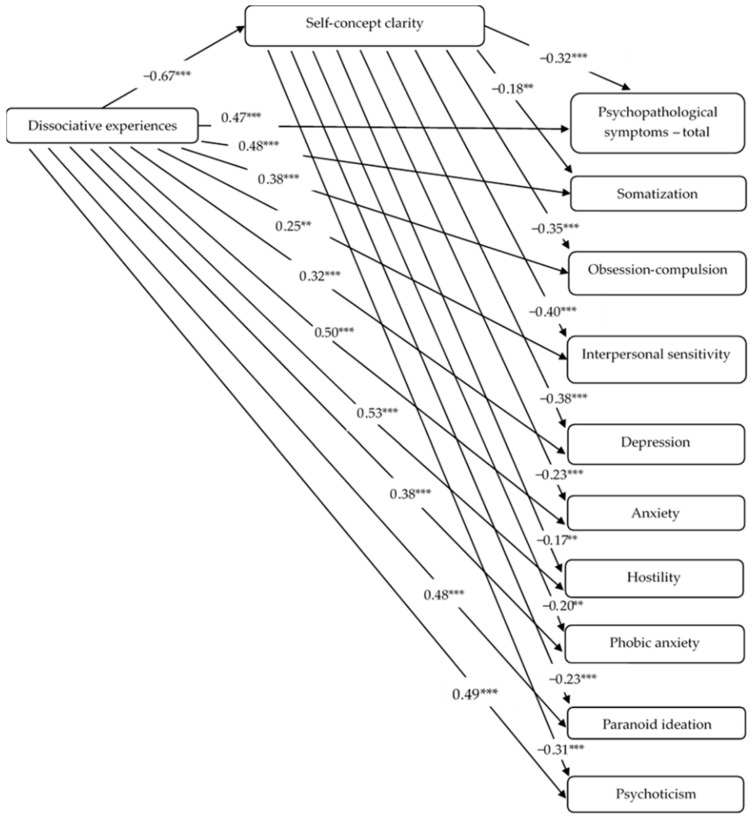
The mediating role of self-concept clarity in the relationship between dissociative experiences and psychopathological symptoms. Note. Standardized coefficients are reported. The figure summarizes results from ten separate simple mediation models. On the path between dissociative experiences and psychopathological symptoms, the value represents the coefficient for the direct association. ** *p* < 0.01; *** *p* < 0.001.

Using the PROCESS extension in SPSS, we analyzed the mediating role of self-concept clarity in the relation between dissociative experiences and psychopathological symptoms (see Figure 1). We computed ten separate models, across which dissociative experiences were the independent variable, self-concept clarity was the mediator, and the outcome variable was either the total SCL-90 score or one of its dimensions. In all the models, participants’ age, gender, and educational level were introduced as control variables. The results showed that the total association of dissociative experiences with psychopathological symptoms (total score) was positive and significant (*β* = 0.68, *p* < 0.001). When considering the individual dimensions of psychopathological symptoms, the results also indicated a positive and significant total association of dissociative experiences with *somatization* (*β* = 0.60, *p* < 0.001), *obsessive–compulsive* (*β* = 0.61, *p* < 0.001), *interpersonal sensitivity* (*β* = 0.52, *p* < 0.001), *depression* (*β* = 0.57, *p* < 0.001), *anxiety* (*β* = 0.66, *p* < 0.001), *anger*–*hostility* (*β* = 0.64, *p* < 0.001), *phobic anxiety* (*β* = 0.52, *p* < 0.001), *paranoid ideation* (*β* = 0.63, *p* < 0.001), and *psychoticism* (*β* = 0.70, *p* < 0.001). In addition, when self-concept clarity was introduced in the model as a mediator, dissociative experiences were positively related to psychopathological symptoms (*β* = 0.47, *p* < 0.001) and all of their dimensions: *somatization* (*β* = 0.48, *p* < 0.001), *obsessive–compulsive* (*β* = 0.38, *p* < 0.001), *interpersonal sensitivity* (*β* = 0.25, *p* = 0.0002), *depression* (*β* = 0.32, *p* < 0.001), *anxiety* (*β* = 0.50, *p* < 0.001), *anger*–*hostility* (*β* = 0.53, *p* < 0.001), *phobic anxiety* (*β* = 0.38, *p* < 0.001), *paranoid ideation* (*β* = 0.48 *p* < 0.001), and *psychoticism* (*β* = 0.49, *p* < 0.001). Moreover, dissociative experiences were negatively associated with self-concept clarity (*β* = −0.67, *p* < 0.001). Further, self-concept clarity was negatively linked to the total score of psychopathological symptoms (*β* = −0.32, *p* < 0.001) and the specific dimensions: *somatization* (*β* = −0.18, *p* = 0.006), *obsessive–compulsive* (*β* = −0.35, *p* < 0.001), *interpersonal sensitivity* (*β* = −0.40, *p* < 0.001), *depression* (*β* = −0.38, *p* < 0.001), *anxiety* (*β* = −0.23, *p* = 0.0003), *anger*–*hostility* (*β* = −0.17, *p* = 0.010), *phobic anxiety* (*β* = −0.20, *p* = 0.005), *paranoid ideation* (*β* = −0.23 *p* = 0.0003), and *psychoticism* (*β* = −0.31, *p* < 0.001). The results of the mediation analysis showed that self-concept clarity mediated the relation between dissociative experiences and the total score of psychopathological symptoms (*β* = 0.21, *SE* = 0.04, 95% CI [0.13, 0.30]). In addition, the results showed the mediating role of self-concept clarity in the relations of dissociative experiences with specific psychopathological symptoms: *somatization* (*β* = 0.12, *SE* = 0.05, 95% CI [0.02, 0.22]), *obsessive–compulsive* (*β* = 0.23, *SE* = 0.04, 95% CI [0.14, 0.33]), *interpersonal sensitivity* (*β* = 0.27, *SE* = 0.04, 95% CI [0.18, 0.36]), *depression* (*β* = 0.25, *SE* = 0.05, 95% CI [0.16, 0.34]), *anxiety* (*β* = 0.15, *SE* = 0.05, 95% CI [0.06, 0.25]), *anger*–*hostility* (*β* = 0.11, *SE* = 0.04, 95% CI [0.03, 0.20]), *phobic anxiety* (*β* = 0.13, *SE* = 0.05, 95% CI [0.03, 0.24]), *paranoid ideation* (*β* = 0.15, *SE* = 0.04, 95% CI [0.07, 0.24]) and *psychoticism* (*β* = 0.21, *SE* = 0.04, 95% CI [0.14, 0.29]) (see Figure 1). In these models, age was not significantly associated with the total score of psychopathological symptoms or any of the specific dimensions, with all *p*s > 0.05. However, there was a negative relationship between gender and *somatization* (*β* = −0.14, *p* < 0.01), *depression* (*β* = −0.12, *p* < 0.05), *anxiety* (*β* = −0.09, *p* < 0.05), and *phobic anxiety* (*β* = −0.13, *p* < 0.05). Also, educational level was negatively associated with *paranoid ideation* (*β* = −0.13, *p* < 0.01) and *psychoticism* (*β* = −0.12*, p* < 0.01). Thus, women reported higher scores on these dimensions.

## 4. Discussion

The first purpose of this study was to examine whether there are gender differences in terms of dissociative experiences, psychopathological symptoms, and self-concept clarity. We also aimed to identify to what extent psychopathological symptoms are associated with dissociative experiences and self-concept clarity in a non-clinical sample, and to explore the mediating role of self-concept clarity in the relation between dissociative experiences and psychopathological symptoms.

Compared to the scores obtained on our non-clinical sample, clinical participants in other studies scored significantly higher on the *Dissociative Experiences Scale* (e.g., Mazzotti et al., 2016). As indicated by Calciu et al. (2024), clinical participants are more likely to report higher scores on detachment, absorption, and compartmentalization than non-clinical participants. Also, clinical samples (e.g., participants with schizophrenia) show lower *Self-Concept Clarity Scale* scores compared to the results of our study and other healthy participants in the control group (Cicero et al., 2016). Last but not least, clinical samples consistently show significantly higher SCL-90 scores than non-clinical samples across multiple studies. For example, Wongpakaran et al. (2011), observed that depression and anxiety were key components that distinguished normal samples from clinical ones. Although the SCL-90 effectively distinguishes between clinical and non-clinical conditions, it should be noted that it is used as a measure of general distress rather than as a tool for differentiating specific diagnoses (Hildenbrand et al., 2015). However, these comparisons between clinical and non-clinical samples should be interpreted with caution, as such differences may be influenced by several uncontrolled factors, including age, cultural context, and temporal differences between samples.

In terms of gender, our results showed that there were no significant differences in terms of dissociative experiences, self-concept clarity, and the total score of psychopathological symptoms. The absence of gender differences may be explained by the influence of transdiagnostic factors and interindividual variability, as well as by methodological limitations, including the unbalanced distribution of participants by gender within our sample. However, previous studies also suggest inconsistent results (Engel et al., 2021; Luoni et al., 2018). First, dissociation is an automatic process of distress regulation that is less influenced by cultural norms regarding emotional expression, which may lead to the same symptoms regardless of gender (Cavicchioli et al., 2021; Luoni et al., 2018). Second, regarding the clarity of self-concept, some studies have not identified significant differences, while others have suggested that this variability is better explained by contextual factors (Jastrzębska & Błażek, 2022). Furthermore, consistent with our findings, previous studies indicate more severe psychopathological symptoms, such as depression, in women than men (B. Carter et al., 2022; Rodríguez-Roca et al., 2021). A possible explanation may be because women experience emotional burdens and chronic stress more intensely, often reporting psychological symptoms, in contrast to men who minimize and hide their distress.

The results of correlation analysis showed a significant and negative association between dissociative experiences and self-concept clarity, which is consistent with previous findings (e.g., Paetzold & Rholes, 2021), and a positive association between dissociative experiences and psychopathological symptoms. This correlation has been identified in other studies in both clinical and non-clinical populations. For example, the study by Steinberg et al. (2005) found that patients with dissociative disorders reported higher scores on the three factors derived from the Symptom Checklist (SCL-90) items: paranoid–psychotic, anxiety–depression and panic–phobia. Regarding the general population, in a study conducted on a sample of students, both depression and anxiety correlated positively with dissociative experiences (Hozoori & Barahmand, 2013). Further, our results underline a significant and negative relationship between self-concept clarity and psychopathological symptoms, which shows consistency with the model of self-concept clarity (Campbell et al., 1996) that supports its negative relationship with neuroticism. Through this model it can be understood that low self-clarity can affect mental health through several mechanisms, such as difficulties in regulating behavior and emotional instability. Essentially, when self-concept clarity is low, emotions are perceived as threatening and, as a result, they become confusing, overwhelming, or difficult to describe, which maintains anxiety and depression. The negative association between age and dissociative experiences, respectively psychopathological symptoms, can be explained based on psychological maturation processes, the development of emotional regulation strategies, and the progressive integration of identity (Conner, 2016; Santoro et al., 2025). As people age, they show less emotional reactivity and use more adaptive coping mechanisms, which reduces the need for dissociation and the overall level of psychopathological symptoms. From this perspective, it appears that the positive association between age and self-concept clarity refers to the fact that, as they become older, individuals develop more stable and coherent representations of themselves, supported by consolidated social roles and an increased capacity for self-reflection (Lodi-Smith & Roberts, 2010).

Regarding the mediation analysis, self-concept clarity mediated the relation between dissociative experiences and the total score of psychopathological symptoms, as well as all of its dimensions: *somatization, obsessive–compulsive*, *interpersonal sensitivity*, *depression*, *anxiety*, *anger*–*hostility*, *phobic anxiety*, *paranoid ideation*, and *psychoticism*. Thus, dissociative experiences are negatively related to self-concept clarity, and the decrease in self-concept clarity is, in turn, associated with an increase in psychopathological symptoms.

This finding suggests that the link between dissociation and mental health is not only direct but also manifests through altered self-concept. In other words, dissociation appears to fragment or destabilize an individual’s self-structure, which reduces their ability to regulate their emotions and behaviors. More specifically, as other studies showed (e.g., Bailer et al., 2017; Robinson et al., 2006), low clarity of self-concept may be related to difficulties in identifying emotions, which is further associated with somatic symptoms. However, there are several studies that have examined psychological outcomes (e.g., depression, anxiety) rather than somatic symptoms. The study conducted by Hertel et al. (2024), showed that reduced clarity of self-concept is associated with increased depression, anxiety, and difficulties in regulating impulse control. Further, previous studies have also suggested that self-related vulnerabilities are associated with obsessive–compulsive symptoms. As a final point, dissociation weakens the clarity of the self by disrupting its development, which can increase vulnerability in relationships, interpersonal suspicion, and contributes to the difficulties in integrating internal and external realities (McEnteggart et al., 2017). These findings, supported by the literature, highlight the fact that clarity of self-concept is a mechanism by which dissociation, a general vulnerability factor, is associated not only with the psychological symptomatology overall, but also with specific components.

Although we tested a mediation model in which self-concept clarity is the mediator, alternative models (e.g., reverse mediation, where psychopathological symptoms may be related to dissociative experiences through self-concept clarity) may also be plausible. However, based on the theoretical framework, we consider our proposed direction to be more consistent with existing evidence. A remarkable theoretical model that can explain our mediation mechanism is the diathesis–stress model of psychopathology (Monroe & Cummins, 2014), which argues that people can have a certain level of predisposing factors (known as diatheses) for any mental illness. In our situation, dissociative experiences trigger a pre-existing vulnerability: the lack of self-concept clarity, and this vulnerability reduces the person’s ability to understand and regulate inner experiences and, implicitly, makes them more susceptible to the development of psychopathological symptoms (e.g., depression, anxiety, paranoid ideation). In addition, previous studies support that the self-concept among young people is not as well integrated as it is among adults and older people (Diehl & Hay, 2011), which validates the study on a sample of students and also emphasizes the importance of increasing self-concept from an early age.

### 4.1. Strengths, Limitations, and Future Directions

Previous studies have consistently shown the positive association between dissociative experiences and psychopathological symptoms (e.g., Bertule et al., 2021; Hozoori & Barahmand, 2013; Prasko et al., 2016). However, prior to our study there was no research investigating whether self-concept clarity may have a mediating role in this relationship. Thus, our study is not limited to simple associations between variables, but tests a mediation model in which multiple psychopathological symptoms are analyzed. Moreover, the results presented few gender differences regarding the main variables.

Still, the study is not without limitations. The first is due to the cross-sectional design. For this reason, it does not provide knowledge about the impact of dissociative experiences on psychopathological symptoms over time. Second, the sample consists mostly of young female participants and this may limit the generalizability of the findings, as well as weaken the importance of the resulting gender differences as it may not appropriately reflect factors that can vary significantly between the genders. Although gender differences were identified regarding somatization and depression, the potential moderating role of gender was not examined. Thus, it cannot be determined whether the relationship between dissociative experiences and psychopathological symptoms differs by gender, which represents an important direction for future research.

A major limitation of the study is the exclusive use of self-reporting instruments, which may introduce shared method variance and potentially inflate associations between variables. However, future studies should consider incorporating multi-method approaches to reduce this bias. Our study also relates to the use of self-reporting to determine the absence of mental disorders. This method may lead to potential classification errors, as participants may not be aware of the existence of certain conditions or may choose not to report them, which could affect the internal validity of the study. In addition, our results are not based on a clinical sample, which diminishes their relevance to therapeutic interventions, and the lack of direct measurement of traumatic experiences limits the interpretation of the mechanisms underlying dissociation. Last but not least, a significant limitation of the study relates to the method used to select participants. The study sample may not be fully representative of the general population, which could introduce potential bias and limit the generalizability of the results. Future research could consider a longitudinal design and a clinical sample to allow a more precise assessment of the factors under study and a better understanding of the long-term impact of dissociative experiences on psychopathological symptoms.

### 4.2. Theoretical and Practical Implications

Despite the limitations, the present study has important clinical and theoretical implications for understanding and preventing psychopathological symptoms. From a theoretical perspective, our results provide evidence for the relation between dissociative experiences and self-concept clarity with psychopathological symptoms. In addition to other studies which only supported that self-concept clarity was significantly associated with fewer psychopathological and dissociative symptoms (Holm & Thomsen, 2017), our study showed that self-concept clarity is not just a passive trait but an active mechanism that mediates the relation between psychological experiences (e.g., dissociation) and psychopathological symptoms.

From a practical standpoint, the results highlight the importance of self-concept clarity as a therapeutic goal that can guide the consolidation or even the reconstruction of a coherent, stable, and conscious self. Therefore, increasing self-concept clarity could be an effective therapeutic target for reducing clinical symptoms caused by dissociative experiences, particularly in contexts such as early trauma, episodes of severe stress, and personality disorders (Adelifard et al., 2023). The strongest clinical example is that Dialectical Behavior Therapy has demonstrated improvements in self-concept clarity in individuals with borderline personality disorder (Roepke et al., 2010).

In addition, interventions such as Schema Therapy could facilitate the integration of fragmented aspects of identity, while narrative techniques and values-centered interventions could support the development of a more coherent identity (Shekouhideh & Ghazanfari, 2025). Furthermore, Cognitive Behavioral Therapy could help clarify the self by restructuring dysfunctional beliefs about oneself (Ruggiero et al., 2018). Thus, assessing and addressing the clarity of the self-concept can provide useful directions in clinical assessment and treatment planning for individuals with dissociative experiences.

## 5. Conclusions

The results of the present study suggest that the relationship between dissociative experiences and psychopathological symptoms is explained by the clarity of the self-concept. More specifically, dissociation may contribute to the development of symptoms by disrupting the coherence and stability of the self, highlighting the role of identity organization in psychological vulnerability, as indicated by the diathesis–stress model.

From a practical perspective, the results support the inclusion of self-concept clarity as a target in assessment, prevention, and intervention, particularly among young people. However, there are substantial limitations, and future studies should use longitudinal designs to test causal relationships and experimental interventions to verify whether improving self-clarity leads to a reduction in symptoms.

Thus, this study indicates the importance of a clear self-concept in young people to reduce the risk of developing symptoms of mental disorders and provide useful information for further study in the field.

## Figures and Tables

**Table 1:** The socio-demographic characteristics of participants (*N* = 257).

Sample Characteristics	*N*	% *
Gender		
Female	179	69.6%
Male	78	30.4%
Residential area		
Urban	123	47.9%
Rural	134	52.1%
Last educational level achieved		
Primary education	1	0.4%
Secondary education	16	6.2%
High school education	120	46.7%
Post-secondary education	9	3.5%
Bachelor’s degree	75	29.2%
Master’s degree	33	12.8%
Doctoral degree	3	1.2%
Physical illness diagnosed in the last year		
Yes	17	6.6%
Chronic illness		
Yes	10	3.9%

* Number (*N*) and percentage (%).

**Table 2:** Descriptive statistics and gender differences in the study variables.

	M	SD	Min	Max	Sk	K	Females (*N* = 179) M (SD)	Males (*N* = 78) M (SD)	*t*
1. Dissociative experiences	2.12	1.77	0	8.75	1.14	1.26	2.17 (1.84)	1.99 (1.61)	0.73
2. Self-concept clarity	3.28	0.74	1.33	4.67	−0.32	−0.74	3.20 (0.77)	3.45 (0.64)	−2.44
3. Psychopathological symptoms—total score	1.10	0.75	0	3.44	0.65	−0.29	1.17 (0.78)	0.93 (0.65)	2.52
4. Somatization	1.18	0.83	0	3.67	0.76	0.19	1.28 (0.86)	0.93 (0.70)	3.47 ***
5. Obsession–compulsion	1.34	0.79	0	4.00	0.48	0.002	1.39 (0.82)	1.19 (0.70)	1.85
6. Interpersonal sensitivity	1.29	0.89	0	3.89	0.55	−0.48	1.36 (0.93)	1.09 (0.77)	2.44
7. Depression	1.26	0.92	0	3.54	0.59	−0.64	1.37 (0.95)	0.98 (0.77)	3.49 ***
8. Anxiety	1.01	0.86	0	3.60	0.91	0.20	1.09 (0.91)	0.81 (0.71)	2.68
9. Hostility	0.93	0.96	0	4.00	1.18	0.78	0.94 (0.94)	0.90 (1.00)	0.27
10. Phobic anxiety	0.64	0.73	0	3.57	1.34	1.22	0.72 (0.76)	0.44 (0.60)	3.18
11. Paranoid ideation	1.20	0.92	0	3.83	0.69	−0.25	1.22 (0.96)	1.13 (0.82)	0.74
12. Psychoticism	0.78	0.72	0	3.20	0.97	0.38	0.81 (0.74)	0.70 (0.65)	1.19

Note. *** *p* < 0.001. *N* = 257.

**Table 3:** Pearson correlation for the study variables.

Variables	(1)	(2)	(3)	(4)	(5)	(6)	(7)	(8)	(9)	(10)	(11)	(12)
1. Dissociative experiences	−											
2. Self-concept clarity	−0.67 **	−										
3. Psychopathological symptoms—total score	0.69 **	−0.65 **	−									
4. Somatization	0.60 **	−0.52 **	0.88 **	−								
5. Obsession–compulsion	0.61 **	−0.61 **	0.88 **	0.795	−							
6. Interpersonal sensitivity	0.55 **	−0.60 **	0.89 **	0.69 **	0.81 **	−						
7. Depression	0.58 **	−0.61 **	0.94 **	0.78 **	0.84 **	0.87 **	−					
8. Anxiety	0.65 **	−0.58 **	0.94 **	0.87 **	0.81 **	0.77 **	0.86 **	−				
9. Hostility	0.64 **	−0.52 **	0.80 **	0.68 **	0.62 **	0.66 **	0.70 **	0.76 **	−			
10. Phobic anxiety	0.51 **	−0.47 **	0.76 **	0.68 **	0.63 **	0.65 **	0.68 **	0.73 **	0.55 **	−		
11. Paranoid ideation	0.64 **	−0.56 **	0.87 **	0.69 **	0.75 **	0.82 **	0.79 **	0.78 **	0.73 **	0.61 **	−	
12. Psychoticism	0.69 **	−0.64 **	0.90 **	0.73 **	0.79 **	0.80 **	0.82 **	0.81 **	0.74 **	0.65 **	0.81 **	−
13. Age	−0.27 **	0.17 **	−0.18 **	−0.12 *	−0.17 **	−0.22 **	−0.19 **	−0.15 *	−0.16 **	−0.11	−0.17 **	−0.13 *

Note. * *p* < 0.05; ** *p* < 0.01. *N* = 257.

## Data Availability

The data presented in this study are available on request from the corresponding author.

## Abbreviations

The following abbreviations are used in this manuscript:

SCL-90	The Symptoms Checklist-90
SCCS	The Self-Concept Clarity Scale
DES	The Dissociative Experiences Scale
M	Mean
SD	Standard deviation
Min	Minimum
Max	Maximum
SK	Skewness
K	Kurtosis
